# Supplementary material for: During Water Stress, Fertility Modulated by ROS Scavengers Abundant in Arabidopsis Pistils
Source: Plants (Basel). 2023 May 31;12(11):2182. doi: 10.3390/plants12112182 (PMC10255272; doi:10.3390/plants12112182)
Supplement: Supplementary file 1 [file plants-12-02182-s001.zip › plants-2360722-supplementary.pdf]

SUPPLEMENTAL DATA

Supplemental Table S1. Primers used in this study.

Primer ID	Sequence (5' to 3')	Purpose
ACT2 qF4	ATTCCAGCAGATGTGGATCTC	qPCR and RT-PCR, internal control
ACT2 qR4	AGCCTTTGATCTTGAGAGCTTAG	qPCR and RT-PCR, internal control
PER17 qF3	TTCCAACATCGATTCACTAAGATC	qPCR
PER17 qR3	CCAATCTGGTCCTCCTGTAAG	qPCR
PER28 qF1	TGGGATCGCGTCTTGTGGTA	qPCR
PER28 qR1	TTAGCCTGCCAGCCAAAGTG	qPCR
PER29 qF1	GCAAACACGTGGCAGACTCT	qPCR
PER29 qR1	TATCGTATGTGCACCCATGATG	qPCR
PER17 F	TGTCTCTTCTTCCCCATCTC	RT-PCR and genotyping
PER17 3UTR	TCTTCTCTTTACTAATGATAATTC	RT-PCR
PER28 F	CGTTTTCTGTTCTACTCTTGC	RT-PCR and genotyping
PER28 3UTR	ATCATCAGAAGCGGAAATTAAG	RT-PCR
PER29 F	AGAATCTACAGCTGCATCATG	RT-PCR and genotyping
PER29 3UTR	ACATTTGATAATTATAAATATACATC	RT-PCR
AT3G42570 F	ATGGAGACAAAGAAAGAAAAG	Cloning

AT3G42570 R	TTATAAATATATACCCATAATG	Cloning
APX4 2F	CTGTTCCTTCCTTCACCAAC	RT-PCR and genotyping
APX4 2R	AGTTTGCTCAGATTGATCCGT	RT-PCR and genotyping
PER17 EcoF	GAATTCAAGTATGTCTCTTCTTCC	pEW201ML construct
201ML R	GAATTCTAGATACAAGCAATACATC	pEW201ML construct
401ML F	AGGCCTTCGAATTCAAGTATGTCTCTTCTTCC	pEW401ML construct
PER17 EcoR	GAATTCAAGATACAAGCAATAC	pEW401ML construct
PER28 StuF	AGGCCTAACAAGATGAAGATTGCAAC	pEW202ML and pEW402ML constructs
202ML R	GAATTCCGTTGAATGCTCTACAATTCG	pEW202ML construct
PER28 EcoR	GAATTCTTTAGTTGAATGCTCTAC	pEW402ML construct
PER29 StuF	AGGCCTATGAAACCAAAGAGCAAAG	pEW203ML and pEW403ML constructs
203ML R	GAATTCCATCAACCTTGTCACACAC	pEW203ML construct
PER29 EcoR	GAATTCAATCAACCTTGTCACAC	pEW403ML construct
PER17 R	AGATACAAGCAATACATCAATAG	Genotyping
PER28 R	ATTCGTCCTGATCTCACCAG	Genotyping
PER29 R	CTTCTAATTACTCCTTCATTCC	Genotyping

LBb1	ATTTCGGAACCACCATCAAAC	Genotyping
Sail_LB	CATAACCAATCTCGATACACC	Genotyping
PER17P F	TTATTGATATCCTCTCTTCTTTTG	*PER17::GUS* construct
PER17P R	ACTTTTTTCTTTTTTGGTGTTGTG	*PER17::GUS* construct
PER28P F	TGATCAGATTCGTTTGGGTC	*PER28::GUS* construct
PER28P R	CTTGTTTTTGTAGAAAGTGTTGC	*PER28::GUS* construct
PER29P F	CATTCGATGTACGTATTACTAG	*PER29::GUS* construct
PER29P R	AACTTTCTCTTTTGTTTGGAATTT	*PER29::GUS* construct

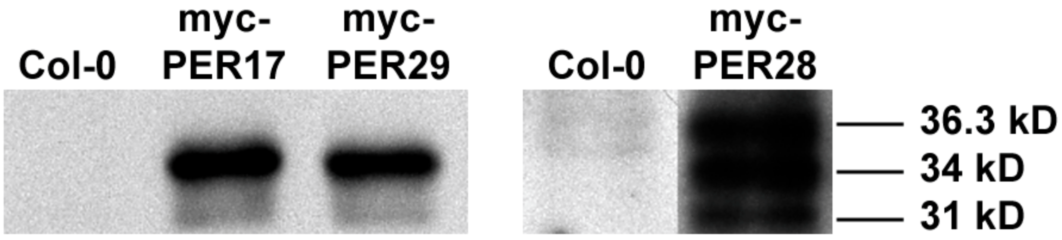

Supplemental Figure S1. Protein expression of myc-PER17 (38.6 kD), myc-PER28 and myc-PER29 (38.4 kD) was detectable using an anti-myc antibody. Other than the predicted 36.3 kD myc-PER28 shown in the immunoblot, two more bands were revealed, possibly due to cryptic splice sites in the myc-*PER28* construct or degradation of this protein during extraction.
